# Restoration of Default Blood Monocyte-Derived Macrophage Polarization With Adalimumab But Not Etanercept in Rheumatoid Arthritis

**DOI:** 10.3389/fimmu.2022.832117

**Published:** 2022-02-23

**Authors:** Audrey Paoletti, Bineta Ly, Samuel Bitoun, Gaëtane Nocturne, Elodie Rivière, Jessica J. Manson, Andrea Matucci, Marc Pallardy, Niek De Vries, Xavier Mariette

**Affiliations:** ^1^Université Paris-Saclay, INSERM UMR1184, Center for Immunology of Viral Infections and Autoimmune Diseases, Le Kremlin Bicêtre, France; ^2^Rheumatology Department, Hôpital Bicêtre, Assistance Publique – Hôpitaux de Paris, Le Kremlin Bicêtre, France; ^3^Department of Rheumatology, University College Hospital, London, United Kingdom; ^4^Department of Rheumatology, Università di Firenze, Firenze, Italy; ^5^Université Paris-Saclay, INSERM, Inflammation Microbiome Immunosurveillance, Châtenay-Malabry, France; ^6^Anti-Biopharmaceutical Immunization: Prediction and Analysis of Clinical Relevance to Minimize the Risk (ABIRISK), Châtenay-Malabry, France; ^7^Amsterdam Rheumatology and Immunology Center, Academic Medical Center of the University of Amsterdam, Amsterdam, Netherlands

**Keywords:** rheumatoid arthritis, monocyte-derived macrophages, M2-like macrophages, Adalimumab, Etanercept

## Abstract

**Introduction:**

We previously reported a specific defect of rheumatoid arthritis (RA) monocyte polarization to anti-inflammatory M2-like macrophages related to increased miR-155 expression in all RA patients except those receiving adalimumab (ADA). In this longitudinal study, we examined whether different tumor necrosis factor inhibitors were able to restore monocyte polarization to M2-like macrophages and their effect on the transcriptomic signature.

**Methods:**

M2-like polarization induced by human serum AB was studied in 7 healthy donors and 20 RA patients included in the ABIRA cohort before and 3 months after starting ADA or etanercept (ETA). The differential gene expression of M2- and M1-related transcripts was studied in macrophage-derived monocytes after differentiation.

**Results:**

At baseline, RA monocytes showed a defect of polarization to M2-like macrophages as compared with healthy donor monocytes, which was negatively correlated with disease activity. M2-like polarization from circulating monocytes was restored only with ADA and not ETA treatment. The transcriptomic signature demonstrated downregulation of M2-related transcripts and upregulation of M1-related transcripts in active RA. In patients receiving ADA, the transcriptomic signature of M2-related transcripts was restored.

**Conclusion:**

This longitudinal study demonstrates that ADA but not ETA is able to restore the M2-like polarization of monocytes that is defective in RA.

## Highlights

- In patients with rheumatoid arthritis, blood monocyte differentiation in anti-inflammatory macrophages is impaired and associated with disease activity.
- This *in vitro* defective blood monocyte polarization to anti-inflammatory macrophages was restored only in patients receiving adalimumab and not etanercept.
- Different types of tumor necrosis factor inhibitors do not have the same effect on monocytes/macrophages.

## Introduction

Monocytes/macrophages are key players in the pathogenesis of rheumatoid arthritis (RA) (1, 2) by secreting tumor necrosis factor α (TNF-α) among other inflammatory cytokines. Synovium tissue macrophages are the most common resident immune cells in the healthy synovial membrane. In active RA with myeloid and lymphoid synovitis, the synovial membrane is leucocyte-rich, including an increased number of pro-inflammatory macrophages. Indeed, besides resident macrophages, blood monocytes can differentiate into monocyte-derived macrophages (MDMs) that may join the synovium and have different phenotypes and functions (3–5).

The “classically activated M1 macrophage phenotype” is considered to be pro-inflammatory, and the “alternatively activated M2 macrophage phenotype” is considered to be regulatory and anti-inflammatory in tissues. Actually, there is a continuum from pro-inflammatory to anti-inflammatory macrophages, with high plasticity between the different states. Classically activated macrophages contribute to RA pathogenesis by secreting pro-inflammatory cytokines and are the main producers of TNF.

We previously showed that RA patients had defective monocyte polarization toward an M2-like macrophage phenotype (CD11b^Lo^–CD71^Lo^; CD206^Lo^; CD163^Lo^ with decreased interleukin 10 [IL-10] secretion) in favor of an M1-like phenotype (inducible nitric oxide synthase^+^, interferon regulatory factor 5^+^, and increased levels of pro-inflammatory cytokines such as IL-1β–IL-6–macrophage inflammatory protein 1α) (6). This defect was specific to RA because it was not found in healthy donors (HDs) or those with other inflammatory diseases (such as Sjogren’s syndrome (SS) and spondyloarthritis (SpA) patients). Moreover, we have also found this specific defect in M2-like polarization of monocytes in RA patients receiving etanercept (ETA) but not adalimumab (ADA).

The objectives of this study were to longitudinally study the roles of the different TNF inhibitors (TNFis) for modifying monocyte polarization in RA patients and to study in detail the macrophage population before and after initiation of ADA treatment.

## Materials and Methods

### Patients

All RA patients fulfilled the 2010 American College of Rheumatology and European League Against Rheumatism RA criteria (7). The Disease Activity Score in 28 joints (DAS28) was used to assess the disease activity of RA patients. Because of the impact of corticosteroids on macrophages, patients receiving corticosteroid therapy ≥10 mg per day were excluded (8). Cells from two different cohorts of patients were obtained:

- Peripheral blood mononuclear cells (PBMCs) from patients in the ABIRA cohort included in the European consortium ABIRISK. This prospective cohort, set up to look for predictors of immunization to biologics, included patients with failure of methotrexate (MTX) and receiving for the first-time ADA or ETA with at least 3-month follow-up (ClinicalTrials.gov: NCT02116504). MTX resistance was defined by disease activity defined by the DAS28 (>3.2, moderate activity; >5.1, high activity) after at least 3 months of MTX. Patients included in the ABIRA cohort were used for phenotyping of monocytes and macrophages before and after anti-TNF treatment.
- Blood MDMs included for RNA-sequencing (RNA-seq) were from patients referred to the Department of Rheumatology of Hôpitaux Universitaires Paris-Sud between September 2017 and May 2019, who all gave their informed consent for use of cells for clinical research. The study was approved by the ethics committee (CPP Sud Méditerranée V2020-A00509-30).

For PBMCs and blood MDMs from HDs, our institutional review board gave their approval for the collection of blood from healthy people (centralized for all French healthy blood donors at Etablissement Francais du Sang in France) under the control of convention with the INSERM.

To compare patients included in the ABIRA cohort, HD PBMCs were frozen under the same condition. Briefly, PBMCs were frozen at 6 to 10.10^6^/ml with autologous plasma–10% dimethyl sulfoxide (DMSO); then cells were placed at −80°C for 48 h and then transferred to −150°C.

### Monocyte Selection, Peripheral Blood Mononuclear Cell Thawing, and Differentiation Into Macrophages

For PBMC thawing, cells were placed in a water bath at 37°C and rapidly transferred to complete Roswell Park Memorial Institute (RPMI). Cells were then washed and counted for monocyte staining and polarization to M2 macrophages, we remove samples under 1.10^6^ PBMCs and viability under 80% of trypan blue. For M2 differentiation with human serum AB (SAB), fresh blood monocytes were isolated by using a pan-monocyte negative selection according to the manufacturer’s instructions (Miltenyi Biotec, Bergisch Gladbach, Germany) to achieve purity of 90% (for RNA-seq) or thawed PBMCs (from patients included in the ABIRA cohort) and then cultured at 1.10^6^ cells/ml for 6 days in hydrophobic Teflon dishes (Lumox; Duthsher) in a macrophage medium (RPMI 1640 medium supplemented with 200 mM of l-glutamine, 100 U of penicillin, 100 μg of streptomycin, 10 mM of HEPES, 10 mM of sodium pyruvate, 50 μM of β-mercaptoethanol, 1% minimum essential medium vitamins, and 1% nonessential amino acids) containing 15% of heat-inactivated human serum AB as a natural source of *M-CSF*, *IL-10*, *IL-4*, and *IL-13*. After 6 days of culture, MDMs were then harvested and suspended in 100% fetal bovine serum to avoid cell death, scratched, and washed with phosphate-buffered saline (PBS) 1×.

### Flow Cytometry

For monocyte subpopulation staining, PBMCs were saturated with FcBlock and incubated for 30 min at 4°C with anti-CD2-CD19-CD56 (PerCP-cy5.5), anti-HLA-DR (FITC), anti-CD16 (APC), and anti-CD14 (Amcyan) antibodies; dead cells were excluded by using fixable live-cells (APC-cy7) (gating strategy in **Supplementary Figure 1A**). For determining macrophage phenotype after monocytes polarization by SAB, cells were harvested in 100% fetal bovine serum and scratched, washed once with phosphate-buffered saline, saturated with FcBlock, and incubated for 30 min at 4°C with anti-CD2-CD19-CD56 (FITC), anti-HLA-DR (AmCyan), anti-CD11b (PerCP-cy5.5), anti-CD71 (PE), anti-CD206 (Pe-cy7), and anti-CD163 (PB) antibodies; dead cells were excluded by using fixable live cells (APC-cy7) (gating strategy in **Supplementary Figure 1B**). The indicated antibodies and isotype-matched antibodies used were obtained from Biolegend (San Diego, Californie). Stained cells were acquired by using a BD FACSCanto II (BD Biosciences, San Jose, CA, USA) and analyzed by using FlowJo V10; gates were defined by using an isotype for each patient.

### RNA-Sequencing and Bioinformatics Analysis

For RNA-seq, after blood monocyte isolation and differentiation into M2-like macrophages with SAB, mRNAs from macrophages were isolated by using the GeneJet RNA purification Kit (Life Technologies, Carlsbad, CA, USA) and QIAshredder (Qiagen, Valencia, CA, USA). The quality of the samples (RNA integrity number [RIN]) was assessed on the Agilent 2100 Bioanalyzer following the manufacturer’s instructions. To construct the libraries, 250 ng of high-quality total RNA (RIN > 8) was processed by using the TruSeq Stranded mRNA kit (Illumina, San Diego, CA, USA) according to the manufacturer’s instructions. Briefly, after purification of poly-A-containing mRNA molecules, mRNA molecules are fragmented and reverse-transcribed with random primers. Replacement of dTTP by dUTP during the second-strand synthesis achieved strand specificity. The addition of a single A base to the cDNA was followed by ligation of Illumina adapters.

Libraries were quantified by a combination of Qubit concentration values and library average size from profiles obtained with the DNA High Sensitivity LabChip kit on the Bioanalyzer 2100. Libraries were sequenced on an Illumina Nextseq 500 instrument using paired-end 75 base-length reads (chemistry v2.5). The average number of reads per sample was 36.7 ± 4.4 million. After sequencing, a primary analysis based on AOZAN software (automated post-sequencing data-processing pipeline) was used to demultiplex and control the quality of the raw data (based on bcl2fastq v2.20.0.422 and FastQC v0.11.5). Obtained fastq files were then aligned by using the STAR algorithm (v2.7.1a). Reads were counted by using RSEM, and the statistical analyses of the read counts involved using the DESeq2 package v1.22.2 (R v3.5.3) to determine the proportion of differentially expressed genes between two conditions. The ingenuity pathway analysis (IPA) database was used to examine the potential functions and regulatory mechanisms of differentially expressed genes in RA-MTX patients versus HDs or RA-ADA versus RA-MTX patients by “upstream regulators” analysis.

### Statistical Analysis

The data were analyzed by using Graph Pad Prism V9.0.1. Data were tested by the Mann–Whitney for two groups and the Kruskal–Wallis test with Dunn’s multiple comparisons for multiple groups, expressed as mean ± SD or SEM with plot individual values. Correlation between variables was tested with two-tailed nonparametric Spearman’s correlation, with a 95% CI. *p* < 0.05 was considered significant.

## Results

### Patients

Twenty RA patients with failure of MTX treatment were included and analyzed before and at 3 months after TNFi initiation (n = 10 ADA; n = 10 ETA). We also included 7 HDs. Nine RA patients were recruited at Bicêtre hospital, and 4 HDs were used for transcriptomic analysis. Their characteristics are in **Table 1**.

**Table 1:** Demographic and clinical data.

A. Healthy donors (HDs) and ABIRA patients with rheumatoid arthritis (RA)		
	HD (n = 7)	RA (n = 20)
Female, n (%)	29	85
Age, mean (SD)	38 (16.5)	55 (23–73)
Disease duration, years, median (range)	–	10 (0–20)
ACPA, n (%)	–	70
RF, n (%)	–	65
DAS28, median (range)	–	4.65 (2.4–5.8)
CRP, mg/ml, median (range)	–	7 (0.09–48)
Co-treatment, n (%)		
MTX		15 (75)
**B. HDs and patients included in RNA-seq analysis**		
	**HD (n = 4)**	**RA (n = 9)**
Female, n (%)	3 (75)	8 (89)
Age, mean (SD)	38.2 (11.9)	57.1 (17.9)
Disease duration, years, median (range)	–	11.5 (3–22)
Anti-CCP, n (%)	–	78
DAS28, median (range)	–	4.24 (1.7–7.1)
Treatment groups		
• MTX	–	5
• ADA	–	3
• IFX	–	1
Co-medications in the ADA/IFX group, n (%)		
Methotrexate		3 (75)
Leflunomide		1 (25)

ACPA, anti-citrullinated protein antibodies; RF, rheumatoid factor; DAS28, Disease Activity Score in 28 joints; MTX, methotrexate; ADA, adalimumab; IFX, infliximab.

### Defective M2 Polarization of Rheumatoid Arthritis Monocytes at Baseline Correlated With Disease Activity

**Figure 1:**
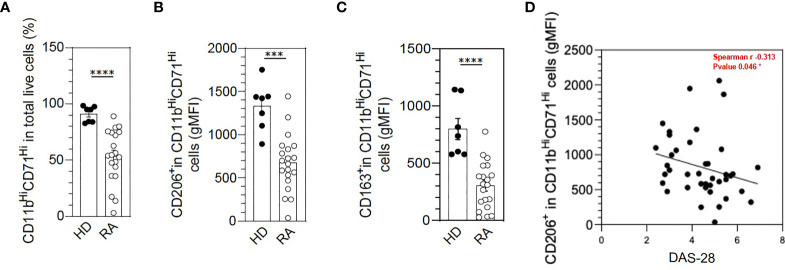
M2-like macrophage polarization defect in rheumatoid arthritis (RA) monocytes under serum AB differentiation. The differentiation of frozen peripheral blood mononuclear cells (PBMCs) to M2-like macrophages was assessed in healthy donors (HDs) (n = 7) and RA patients (n = 20) by flow cytometry with anti-CD11b and anti-CD71 antibodies **(A)**. Specific markers of M2-like macrophage polarization were assessed: CD206 **(B)** and CD163 **(C)**. Spearman’s correlation analysis of macrophage CD206 expression in RA patients (geometric mean [gMFI]) and disease activity (Disease Activity Score in 28 joints [DAS28]) **(D)**. Data are shown as symbols and mean ± SEM and were compared by Mann–Whitney t-test. ***p < 0.001 and ****p < 0.0001.

At baseline, we confirmed that in RA patients, macrophages differentiated from monocytes with human SAB showed significantly fewer cells with the pan-macrophage markers CD11b and CD71 (*p* = 0.0005) as compared with HDs (**Figure 1A**) and fewer cells with expression of M2-like markers such as CD206 (*p* = 0.008) and CD163 (*p* = 0.0003) (**Figures 1B, C**). We found a negative correlation (Spearman = −0.313) between disease activity, measured by the DAS28, and the number of M2-like CD206^+^ macrophages (**Figure 1D**), Thus, patients with a higher monocyte defect of polarization in M2-like macrophages had higher disease activity.

### Defective M2 Polarization of Rheumatoid Arthritis Monocytes Is Restored After 3 Months of Adalimumab But Not Etanercept

**Figure 2:**
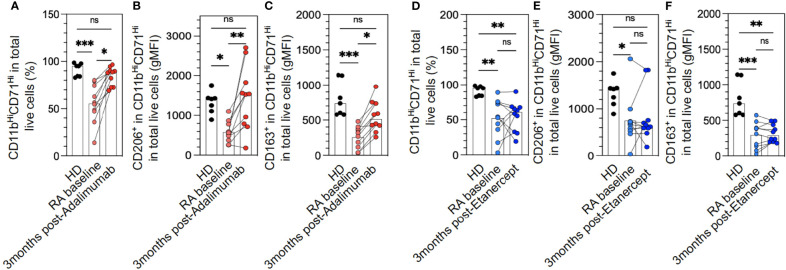
M2-like macrophage polarization defect in rheumatoid arthritis (RA) monocytes is restored with adalimumab (ADA) but not etanercept (ETA) treatment. The differentiation of frozen peripheral blood mononuclear cells (PBMCs) to M2-like macrophages was assessed in healthy donors (HDs) (n = 7) and RA patients at baseline and divided upstream between patients who would receive ADA **(A–C)** or ETA **(D–F)** (n = 20), RA at 3 months after ADA (n = 10, red dots), and RA at 3 months after ETA (n = 10, blue dots) by flow cytometry analysis using anti-CD11b and anti-CD71 antibodies **(A, D)**. Specific markers of M2-like macrophage polarization were assessed: CD206 **(B, E)** and CD163 **(C, F)**. Results are shown as symbols, lines, and mean ± SEM and were compared by Kruskal–Wallis test with Dunn’s multiple comparisons. *p < 0.05, **p < 0.01, and ***p < 0.001. ns, not significant.

Longitudinal follow-up of these patients at 3 months after ADA treatment showed that the M2-like polarization was restored to levels similar to HDs for pan-macrophage markers and CD206 (**Figures 2A, B**). ADA was only partially effective in restoring CD163 levels in macrophages (**Figure 2C**). Conversely, ETA was unable to reverse the M2-like polarization defect (**Figures 2D–F**). Unfortunately, we are not able to demonstrate any correlation between M2-like markers at baseline (such as CD206 or CD163) and clinical or EULAR response at 1, 3, 6, or 12 months after anti-TNF treatment.

### Monocyte Subpopulations and Activation Status Do Not Account for Adalimumab-Induced Rescue of the Polarization Defect

**Figure 3:**
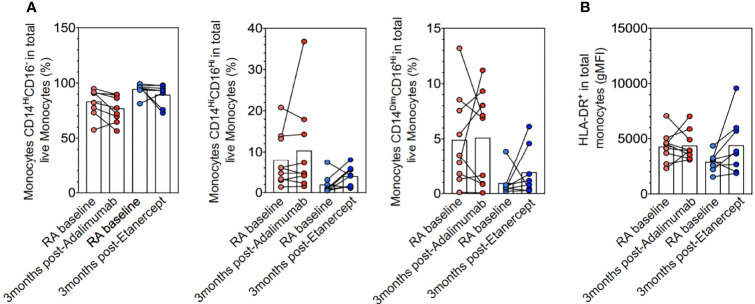
Monocyte subpopulations and activation. *Ex vivo* CD14 and/or CD16 monocytes **(A)** and HLA-DR **(B)** expression in rheumatoid arthritis (RA) at baseline (n = 17), RA at 3 months after adalimumab (ADA) (n = 9), and RA at 3 months after ETA (n = 8) determined by flow cytometry analyses of frozen peripheral blood mononuclear cells (PBMCs with anti-HLA-DR, anti-CD14, anti-CD16, anti-CD2, anti-CD19, and anti-CD56 antibodies. Results are shown as symbols, lines, and mean ± SEM and were compared by Kruskal–Wallis test with Dunn’s multiple comparisons and the two-tailed nonparametric test.

The polarization defect of monocytes to macrophages might be due to the abnormal distribution of monocyte subpopulations or their degree of activation. However, longitudinal follow-up of monocytes (CD14^hi^CD16^−^, CD14^hi^CD16^hi^ and CD14^dim^CD16^high)^) did not significantly differ before and after treatment with ETA or ADA or between ETA and ADA, which suggests that the correction of the defect of M2-like polarization by ADA was not due to a change in the distribution of the monocyte subsets (**Figure 3A**). The expression of HLA-DR was also unchanged after TNFi therapy (**Figure 3B**).

### RNA-Sequencing Analysis Demonstrated the Pro-Inflammatory Endotype of Rheumatoid Arthritis Monocyte-Derived Macrophages and Rescue by Adalimumab

To further analyze in detail the M2-like polarization defect and the role of ADA in reversing it, we used transcriptomic profiling of a transversal set of MDMs from 4 HDs and 9 RA patients. We separated monocytes from RA patients who received MTX alone (n = 5; 4 of them non-responders) and those who received monoclonal anti-TNF antibodies (ADA or infliximab [IFX]) (n = 4; 2 of them non-responders).

Unsupervised IPA of differentially expressed genes between MTX-treated RA patients versus HDs revealed a significant (*p* < 0.05) enrichment of 69 canonical pathways. The top 10 enriched pathways were related to ERK/MAPK, PI3K/AKT, STAT3, or granulocyte-macrophage colony-stimulating factor (GM-CSF) signaling (**Supplementary Figure 2A**).

**Figure 4:**
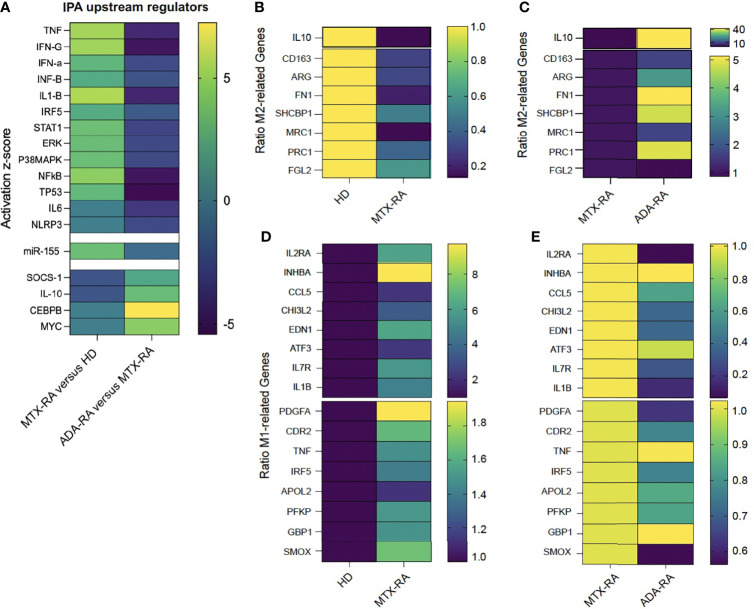
The defect in M2-like polarization leads to an M1-like macrophage phenotype. Heatmap of a predicted upstream regulator effect on M2-like macrophages after monocyte polarization with serum AB (SAB) differentiation in rheumatoid arthritis-methotrexate (RA-MTX) patients versus healthy donors (HDs) or RA-adalimumab (RA-ADA) versus RA-MTX patients. The color of each square represents the activation z-score: activated = yellow (+5), inhibited = dark blue (−5) **(A)**. Heatmap of the ratio of the normalized count of M2-related genes in RA-MTX patients versus HDs (HD = 1 yellow) **(B)** or RA-ADA patients versus RA-MTX patients (RA = 1 dark blue) **(C)**. Ratio of normalized counts of M1-related genes in RA patients versus HDs (HD = 1 dark blue) **(D)** or RA-ADA patients versus RA patients (RA = 1 yellow) **(E)**. MRC-1 gene = CD206 receptor.

This observation was further complemented by the enrichment of several pro-inflammatory cytokines or transcription factors involved in macrophage polarization, such as TNF-IFN-γ/α/β-IL-1β-IRF5-TP53-STAT1-ERK-p38MAPK-NFκB, and inhibition of IL-10-SOCS-1 (*p* < 0.05) (**Figure 4A**), as top upstream regulators by IPA. Interestingly, miR-155 was the most upregulated miR in MTX-treated RA patients versus HDs (**Figure 4A**). ADA/IFX-treated RA patients showed inhibition of a pro-inflammatory macrophage response and activation of an anti-inflammatory response, such as the factors SOCS-1, IL-10, CEBPβ, and c-MYC (9) (**Figure 4A**). There was no statistical difference in miR-155 expression between MTX-treated and ADA/IFX-treated RA patients (**Figure 4A**).

Then, we performed a supervised analysis looking at genes associated with macrophage polarization. The terminology and markers to describe macrophage activation are heterogeneous; to address obstacles in describing macrophage activation, we selected the papers of Murray et al., Locati et al., and Sica et al. to choose M2 and M1 markers (10–12).

We defined a set of 8 M2- and 16 M1-associated genes (10–12). The 8 M2-related genes were downregulated in MTX-treated RA as compared with HDs (**Figure 4B**), and the 16 M1-related genes were upregulated (**Figure 4D**). Conversely, as compared with MTX-treated RA patients, in ADA/IFX-treated RA patients, 7 M2-related genes were upregulated (**Figure 4C**) and 12 M1-related genes were downregulated (**Figure 4E**).

Finally, IPA of RA patients receiving ADA/IFX and those receiving no TNFi confirmed, according to the top diseases and functions, a decrease in rheumatic disease (activation score −2.2, 89 molecules related) and inflammation of joints (activation score −2.3, 69 molecules related) (**Supplementary Figure 2B**).

## Discussion

In RA patients with failure of MTX who were enrolled in the ABIRA study, we confirmed specific abnormalities of monocyte polarization in anti-inflammatory macrophages. The proportion of anti-inflammatory macrophages was negatively correlated with disease activity. This defective monocyte polarization in anti-inflammatory macrophages was reversed by ADA but not ETA treatment. The transcriptomic analysis confirmed that stimulation in HD orientates to an anti-inflammatory macrophage phenotype, which leads to RA to a few anti-inflammatory macrophages and a majority of pro-inflammatory macrophages, which was reversed by ADA treatment.

The link between the proportion of blood monocyte-derived anti-inflammatory macrophages and disease activity supports the importance of this regulatory population even outside the joint. Previous studies suggested that synovial macrophages (SMs) have two major origins, namely, tissue-resident and monocyte-derived SMs, where the pro-inflammatory-M1 or anti-inflammatory-M2 concept does not reflect the phenotype of SMs (13, 14). The complexity of synovial tissue macrophages (STMs) leads to several clusters of macrophages (13–15), and Alivernini et al. demonstrated an increase in the population of CD163^pos^CD206^pos^ STMs in patients in remission who had received TNFi and MTX. Moreover, the amount of CD163^pos^CD206^pos^ STMs is higher in sustained RA remission than in patients with flare (15).

This study has some limitations. The number of patients followed up longitudinally was low (10 with each drug), the duration of disease was heterogeneous, and age and sex differ between HDs and RA patients. The transcriptomic analysis was not performed in patients who received ETA. The choice of the M1- and M2-related genes may be the subject of discussion, we used only CD206 and not CD163 for defining M2-like macrophages and we did not provide functional experiments.

Despite these limitations, this study sheds some light on a possible different mechanism of action of the different types of TNFi. With a longitudinal 3-month follow-up, we showed a restoration of M2-like anti-inflammatory macrophages with ADA but not ETA treatment. Monoclonal anti-TNF antibodies (such as ADA/IFX) and the TNF receptor 2 (TNFR2)-immunoglobulin (ETA) can bind soluble TNF and membrane TNF (mTNF), but the stability of the interaction with mTNF is higher, with monoclonal anti-TNF antibody revealing a possible differential mechanism of action of these molecules (16). Our study shows that monocyte polarization could be used as a biomarker to help to choose the best anti-TNF in RA patients. In case of a defect of polarization of monocytes into anti-inflammatory M2-like macrophages, ADA could be preferred over ETA. In addition, the assessment of polarization could be used as a biomarker for predicting and following the efficacy of anti-GM-CSF treatment, a new and very promising drug in RA. Indeed, anti-GM-GSF is able to inhibit the differentiation of monocytes into pro-inflammatory M1-like macrophages and thus can reverse the defect of polarization into anti-inflammatory M2-like macrophages.

Thus, if TNFi in RA acts mainly by inhibiting soluble TNF and if this action is the same between both types of drugs, there might be a supplementary action of monoclonal anti-TNF antibody by binding mTNF, which is mainly expressed by monocytes/macrophages. Some examples of a differential action of TNFi have been published regarding the activation of T regulatory cells specific to the monoclonal anti-TNF antibody *via* an increase in mTNF level and direct stimulation of T regulatory cells *via* TNFR2 (17). Recently, Diallo et al. demonstrated in mice transgenic for TFNR1−/−, TNFR2−/−, and tmTNFKI/KI (3TG mice), with canonical TNF signaling abolished and soluble TNF not secreted by monocytes/macrophages, that M1 macrophages polarized from monocytes of bone marrow and treated by an anti-murine-TNF antibody (MP6-XT22) or ETA inhibited the expression of pro-inflammatory cytokines and inducible nitric oxide synthase mainly by upregulating arginase 1 (18).

The mechanism of the restoration of M2-like macrophage differentiation by monoclonal anti-TNF antibody remains to be explored. Our transcriptomic study clearly showed that monocytes could not differentiate from anti-inflammatory macrophages. The link between the binding to mTNF and this defect is still unknown. In our previous study, we found that miR-155 was overexpressed in RA monocytes and M2 macrophages except in ADA-treated patients and could lead to this defect when miR-155 was introduced in healthy monocytes. In this study, we confirm that miR-155 is increased in M2-like macrophages from MTX-treated RA but also in M2-like macrophages from ADA/IFX-treated patients.

In conclusion, our study demonstrated that ADA but not ETA could restore the M2-like polarization of monocytes that is defective in RA. This is another example of the differential action of the two types of TNFi. The pathway involved in this restoration should be further studied to identify novel therapeutic targets.

## Data Availability Statement

The data discussed in this publication have been deposited in NCBI's Gene Expression Omnibus (19) and are accessible through GEO Series accession number GSE196743 (https://www.ncbi.nlm.nih.gov/geo/query/acc.cgi?acc=GSE196743).

## Ethics Statement

The studies involving human participants were reviewed and approved by CPP SudMéditerranée V2020-A00509-30 for patients included in the RNA-Seq Analysis and ClinicalTrials.gov:NCT02116504 for patients included in the ABIRA cohort. The patients/participants provided their written informed consent to participate in this study.

## Author Contributions

AP and BL performed all experiments. AP, SB, GN, and XM contributed to the conception and design of the study and wrote the manuscript. NDV organized the ABIRA sample storage. All authors contributed to the manuscript revision and read and approved the submitted version.

## Funding

This work was supported by the Laboratoire d’Excellence en Recherche sur le Médicament et l’Innovation Thérapeutique (ANR10), Fondation pour la Recherche Médicale DEQ20150934719, ABIRISK (Anti-Biopharmaceutical Immunization: prediction and analysis of clinical relevance to minimize the risk), and the Innovative Medicines Initiative Joint Undertaking (grant no. 115303), resources of which are composed of financial contribution from the European Union’s Seventh Framework Programme (FP7/2007-2013) and EFPIA companies’ in-kind contribution.

## Conflict of Interest

XM has served on scientific advisory boards for BMS, Galapagos, GSK, Janssen, Novartis, Pfizer, and UCB.

The remaining authors declare that the research was conducted in the absence of any commercial or financial relationships that could be construed as a potential conflict of interest.

## Publisher’s Note

All claims expressed in this article are solely those of the authors and do not necessarily represent those of their affiliated organizations, or those of the publisher, the editors and the reviewers. Any product that may be evaluated in this article, or claim that may be made by its manufacturer, is not guaranteed or endorsed by the publisher.

## Abbreviations

RA, rheumatoid arthritis; ADA, adalimumab; ETA, etanercept; IFX, infliximab; HD, healthy donor; M1-like, classically activated macrophage phenotype; M2-like, alternatively activated macrophage phenotype; SAB, human serum AB; ABIRISK, Anti-Biopharmaceutical Immunization prediction and analysis of clinical relevance to minimize the RISK; ABIRA, Anti-Biopharmaceutical Immunization in Rheumatoid Arthritis; DAS28, Disease Activity Score in 28 joints; IPA, ingenuity pathway analysis; MTX, methotrexate; TNFi, tumor necrosis factor inhibitor.
