# Supplementary material for: Restoration of Default Blood Monocyte-Derived Macrophage Polarization With Adalimumab But Not Etanercept in Rheumatoid Arthritis
Source: Front Immunol. 2022 Feb 23;13:832117. doi: 10.3389/fimmu.2022.832117 (PMC8904384; doi:10.3389/fimmu.2022.832117)
Supplement: Supplementary file 1 [file DataSheet_1.docx]

Supplementary Material

# Supplementary Figures and Tables

***
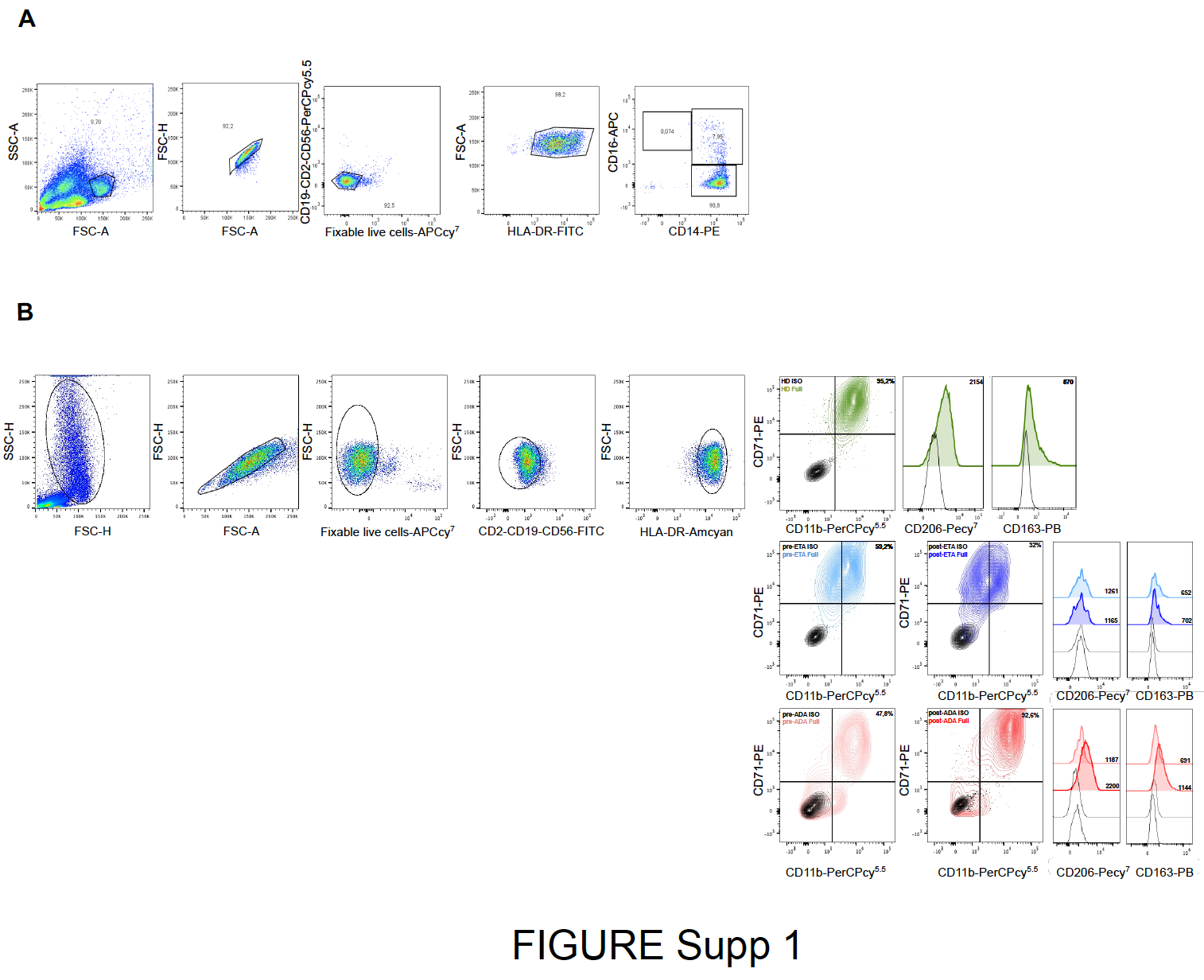
***

***Supplemental Figure 1: M2-like macrophage polarization under SAB differentiation and monocyte gating strategies.*** **(A)** Gating strategy used for monocyte subsets on PBMCs. **(B)** Gating strategy used to determine monocyte polarization into M2-like macrophages after 6 days’ culture on SAB.


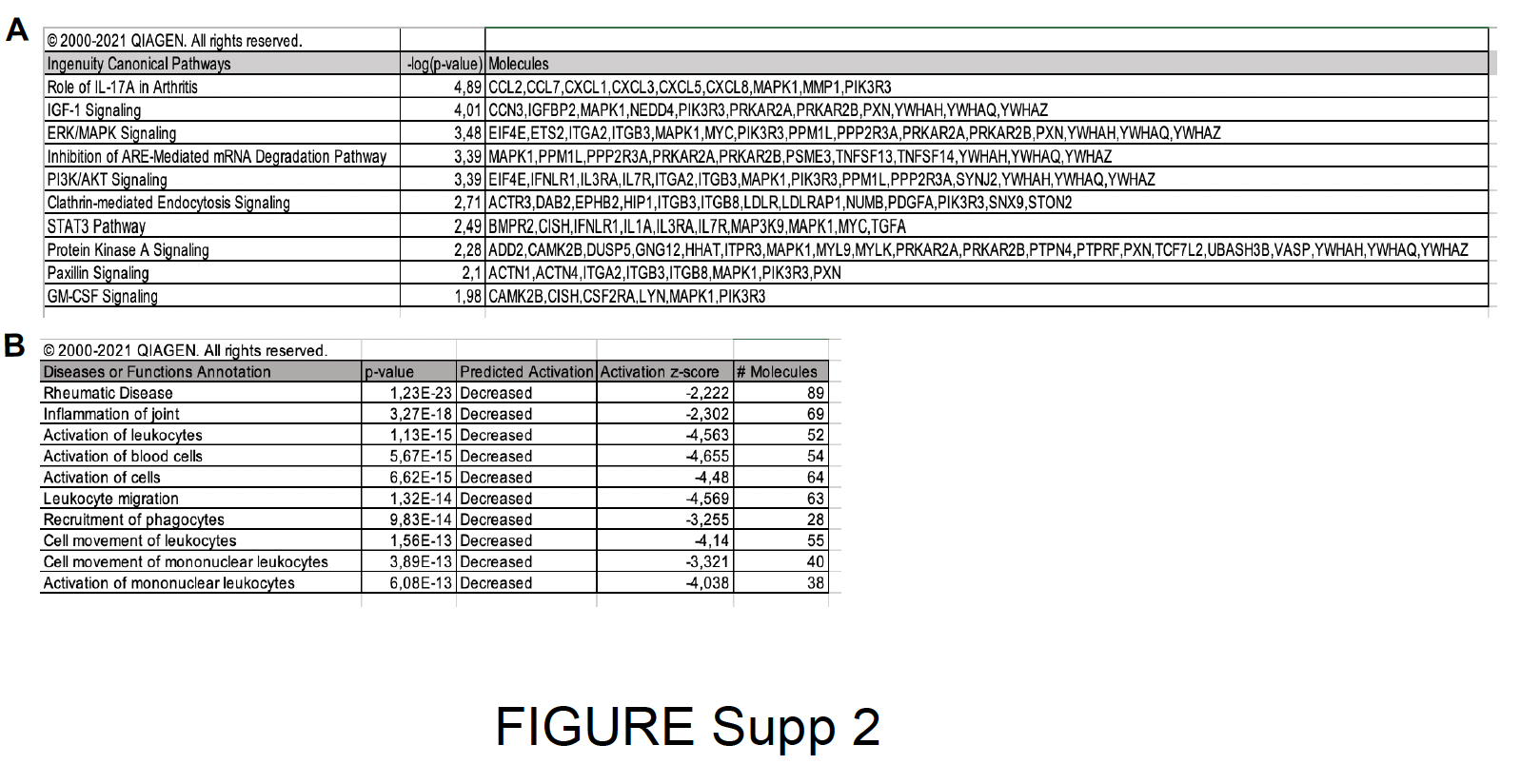


***Supplemental Figure 2: Ingenuity pathway analysis.* (A)** Top 10 enriched canonical pathways between MTX-treated RA patients versus HDs. **(B)** Top 10 diseases and functions of ADA/IFX-treated RA patients as compared with RA patients not treated with tumor necrosis factor inhibitors.
